# Characterization of the variations in the industrial processing and nutritional variables of poultry by-product meal

**DOI:** 10.1016/j.psj.2022.101926

**Published:** 2022-04-22

**Authors:** Josiane Aparecida Volpato, Leonir Bueno Ribeiro, Guilherme Baú Torezan, Ingrid Caroline da Silva, Isabela de Oliveira Martins, Jansller Luiz Genova, Newton Tavares Escocard de Oliveira, Silvana Teixeira Carvalho, Paulo Levi de Oliveira Carvalho, Ricardo Souza Vasconcellos

**Affiliations:** ⁎ Animal Science Department, State University of Western Paraná (Unioeste), Marechal Cândido Rondon, PR 85960-000, Brazil; † Animal Science Department, State University of Maringá, Maringá (UEM), PR 87020-900, Brazil; ‡ Animal Science Department, Federal University of Viçosa (UFV), Viçosa, MG 36570-900, Brazil

**Keywords:** animal meal, monitoring, process standardization, animal protein, nutritional quality

## Abstract

The study aimed to measure variations in industrial process and nutritional variables of poultry by-product meal (**PBM**) in rendering plants from batch cookers. A total of 200 samples of low ash PBM with mineral matter (**MM**) content of 11% (LA, n = 104) or high ash with MM above 11% (HA, n = 96) were collected from 5 industrial processing plants. The highest coefficients of variation in chemical composition were for MM (LA - 19.70%; HA - 19.59%), ether extract (LA - 20.72%; HA - 14.86%), collagen (LA - 21.16%; HA - 30.00%) and water activity (LA - 24.54%; HA - 25.89%). However, the crude protein (LA - 5.07%; HA - 7.39%), dry matter (LA - 1.75%; HA - 2.90%) and organic matter digestibility (LA - 4.81%; HA - 6.78%) were lower. The variability of the data related to the process of PBM was: maximum process temperature (LA - 3.91%; HA - 3.56%), average process temperature (LA - 3.73%; HA - 4.71%), and processing time (LA - 27.37%; HA - 37.59%). This study evidenced that the corrective measures by limiting the amount of bones in the raw material, optimizing the pressing step for the poultry fat extraction, and also controlling the processing time of PBM may favor the production of more standardized PBM in terms of chemical composition and quality.

## INTRODUCTION

The production of poultry as a source of animal protein in human food is responsible for the generation of large amounts of by-products. In 2020 more than 5 billion poultry were slaughtered for meat production in Brazil (IBGE, 2020) and approximately 25% of the total slaughtered is not used for human consumption (ABRA, 2019). In the processing step of these poultry by-products, several important nutrients such as amino acids, fatty acids, and minerals add a high nutritional value to the meals (Ribeiro et al., 2019).

In this way, these ingredients present an important contribution to industrial ecology, in which waste from one step of the production chain is used as a source of raw materials for a subsequent step (rendering facilities) and contributes substantially to reducing the environmental impacts of the sector (Jayathilakan et al., 2012; Zagklis et al., 2020).

The main limitations of use of poultry by-product meal are related to their quality due to being raw materials susceptible to oxidation process (Laflamme et al., 2014) and microbiological contamination (Jones-Ibarra et al., 2017), besides presenting wide variability in nutritional composition. Regarding this last-mentioned aspect, these by-products are made from a constant flow of waste, making it difficult to segregate the fresh materials from the slaughterhouse to maintain stability in chemical composition, which results in wide variability in nutritional content. The characteristics of the raw materials used for the production of poultry by-product meal and the industrial processing conditions may be the main aspects that negatively affect the digestibility, the wide variations in nutritional composition and the shelf life of this ingredient (Odeyemi et al., 2020).

The quality assurance of food products is associated with the processing conditions and industries attempt to improve these conditions with emphasis on research and development associated with the use of equipment to standardize process steps, using statistical modeling, average value analysis, and variability of experimental data as key point (Engel, 1992). In addition, there are investigations in search of designing equipment in order to optimize processing conditions with emphasis on the final quality of the finished product (Farmanesh et al., 2019). Despite this concern, in Brazil, the industrial facilities of animal by-product plants still lack many investments in equipment and industrial automation in search of improvements in the process of these ingredients.

In view of the importance of knowing the variation existing in industrial processes of ingredients, with the purpose of proposing future improvements in reducing these variations, a study was conducted by compiling and analyzing industrial production data from poultry by-product plants. Therefore, this study aimed to measure variations in industrial process conditions and in the chemical and physical properties and in vitro digestibility of poultry by-product meal in rendering plants.

## MATERIALS AND METHODS

**Figure 1:**
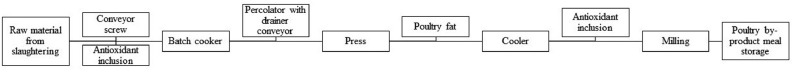
Production diagram of the raw material receiving plants.

At the beginning of the study, processing data for analysis and PBM samples from five establishments related to poultry by-product meal (**PBM**) were collected, in the State of Paraná, Brazil. All selected establishments were inspected by the Brazilian Federal Inspection Service and met production and quality standards (MAPA, 2010). The production diagram of the establishments consisted of the reception step of the raw material (Figure 1); storage in the raw material silo (hopper) until the time of processing; processing in the cooker; removal of the material from the cooker to the percolator and separation of excess oil; transport of the hot cake from the percolator to the press; pressing for the extraction of oil and obtaining the PBM; grinding of PBM (in some places preceded by cooling), application of antioxidants and anti-salmonella and storage of PBM in silos.

### Sampling, Processing, and Classification

A total of 200 PBM samples from 5 different PBM were collected and the establishments were defined as linked (n = 150 samples) and collector (n = 50 samples). Linked establishments (n = 3) are those that have direct communication with the poultry slaughterhouse and the visceral material is transported by pipeline to the processing site. The materials constituting the PBM are conducted continuously to the PBM, with few minutes between the slaughter and its arrival to the waiting silos for the process. Collecting establishments (n = 2) are those with longer distances from the slaughterhouse, without direct communication. The transportation of the raw material to be processed to these establishments is mostly done by road. In overall, the waiting time for this material to be processed is at least 4 h post-slaughter, and can be legally up to 24 h (MAPA, 2010; Meeker and Meisinger, 2015).

Each PBM sample came from a batch production at the PBM. Data regarding the raw material, processing and PBM were recorded for later statistical analysis. The PBM samples were composed of poultry slaughterhouse waste, such as head, feet, digestive tract, respiratory tract, reproductive tract, visceral fat, shavings, skin, cuticles, non-intentionally added feathers, and whole carcasses or part thereof that were rejected for human consumption and mechanically separated meat waste (**MSMW**). Samples with less than 11% mineral matter were classified as low ash (LA, PBM_LA_) and equal to or greater than 11% as high ash (HA, PBM_HA_).

### Data Collection and Analytical Procedures

Information related to the industrial processing (cooking step in the cookers) and to the finished product, that is, the PBM, was recorded. In the cooker processing step, the cooker capacity was measured; oil:viscera ratio added for frying; average process temperature; maximum process temperature and processing time. The processing time was the interval between the start of thermal processing and the output of the cooker material to the percolator. The average and maximum process temperature was determined by the sensors installed in the cookers at 15-min intervals.

In the finished product, data on water activity (**WA**), moisture, dry matter (**DM**), mineral matter (**MM**), organic matter (**OM**), crude protein (**CP**), ethereal extract (**EE**) by acid hydrolysis (**EEAH**), collagen, and in vitro digestibility were collected.

The chemical composition of the PBM samples was performed following the methodologies described by the Association of the Official Analytical Chemists (AOAC, 2005) in which moisture (method 930.15), DM, MM (method 942.05), OM, CP (method 954.01), EEAH (method 954.02), and in vitro digestibility were quantified. For collagen analysis used the method proposed by Ramos and Gomide (2017). For the determination of WA used specific equipment (Pawkit - Decagon, WA). The analyses were performed in the Laboratory of Food Analysis and Animal Nutrition (LANA-UEM, Maringá, PR, Brazil).

Cooker's fill rate was estimated by dividing the weight of raw material fed into the cooker by its total capacity. The oil:viscera ratio added for frying was estimated by dividing the amount of oil included in the process and the weighed amount of viscera in the cooker.

To determine the nutritional quality of PBM, in vitro digestibility analysis was performed. The in vitro digestibility coefficients of OM (**IVDOM**) were determined by adapting the method proposed by Hervera et al. (2007), using 2-compartment model (simulations of stomach and small intestine), with a reduction in the amount of sample from 0.75 g to 0.50 g. This adaptation was made according to the amount of protein substrate to be digested, aiming to ensure that the amount of enzyme present in the medium was adequate to digest the CP of the material.

### Statistical Procedures

Initially, data on the variables of chemical composition, nutritional quality, and processing of the PBM were subjected to descriptive statistic performed on the mother and daughter samples consisting of estimates of the population average from 1,000 bootstrap samples created from the original sample, obtained with replacement (Efron and Tibshirani, 1993).

The estimates of skewness (**sk**), mean (**m**), and standard deviation (**SD**) in the original sample $\left(s{\hat{k}}_{\text{LA}},\;\;s{\hat{k}}_{\text{HA}},\;{\hat{m}}_{\text{LA}},\;\;{\hat{m}}_{\text{HA}},\;\;s_{\text{LA}}\;\;e\;s_{\text{HA}}\right)$ and the mean value estimates of the 1,000 bootstrap samples $\left({s\hat{k}}_{\text{LA}}^{*},\;\;{s\hat{k}}_{\text{HA}}^{*},\;{\hat{m}}_{{}_{\text{LA}}}^{*},\;\;{\hat{m}}_{{}_{\text{HA}}}^{*},\;\;s_{{}_{\text{LA}}}^{*}\;\;{e\;s}_{{}_{\text{HA}}}^{*}\right)$ were calculated to compute the bias of the mean $\left(B_{\text{LA}}^{\hat{m}}\;\;{eB}_{\text{HA}}^{\hat{m}}\right)$ and the standard deviation ($B_{\text{LA}}^{s}{\;e\;B}_{\text{HA}}^{s}$), obtained by difference between the estimates in the mother sample and the respective mean value obtained from the bootstrap sample of mean values.

Sequentially, in both samples the W test for normality (Shapiro and Wilk, 1965) and the asymmetry test for normality were performed, with *P*-value computed by Monte Carlo approximation of the bootstrap distribution (Shapiro et al., 1968). Bias of means was verified by the right-hand tail *t* test for one-sample, with null hypothesis (H_0_) $\mu_{\text{LA}\;}{=\;\mu }_{\text{LA}}^{*}\;\text{or}\;\mu_{\text{HA}\;}{=\;\mu }_{\text{HA}}^{*}$. Standard deviations bias was assessed by the right-hand tail chi-square test for one-sample variance $\left(H_{0}{:\;\sigma }_{\text{LA}}^{2}=\;{\left(s_{\text{LA}}^{*}\right)}^{2}\;\text{or}\;H_{0}{:\;\sigma }_{\text{HA}}^{2}=\;{\left(s_{\text{HA}}^{*}\right)}^{2}\right)$.

If the data showed normality and absence of bias and asymmetry (*P >* α), the variability of the variables was assessed by estimates of confidence intervals (**CI**) of the population mean (µ), with a 95% CI, using Student's t statistics. The estimation of the CI of the mean of µ was performed by the bias-corrected percentile bootstrap (BCBP) method when mean or standard deviation bias was detected (*P ≤* α) in the data (Efron and Tibshirani, 1986). Otherwise, when asymmetry occurred (*P ≤* α), amplitude estimation for µ was performed using the percentile bootstrap method with accelerated bias-corrected (BCa) (Efron and Tibshirani, 1986; Efron and Tibshirani, 1993), computed using the bootstrap package of the R Core Team program.

For samples with CI of µ estimated by t-statistic or BCBP, the comparison between means of PBM types was performed by the *t* test for the CI of the difference between means (CI_DIF_), using the t-statistic. If the CI of µ was estimated by BCa in at least one of the samples, the contrast between means was verified by the U test of the sum of ranks with continuity correction (Mann and Whitney, 1947).

The results according to the establishment were presented as descriptive statistics. The significance level (α) of 0.05 was adopted in all hypothesis tests. The analyses were performed using R Core Team software.

## RESULTS

The population averages were obtained through the bootstrap procedure, as well as their respective standard deviations and bias for chemical composition and digestibility (Table 1). The following coefficients of variation were obtained for DM (LA - 1.75%; HA - 2.90%), MM (LA - 19.70%; HA - 19.59%), CP (LA - 5.07%; HA - 7.39%), EE (LA - 20.72%; HA - 14.86%), WA (LA - 24.54%; HA - 25.89%), IVDOM (LA - 4.81%; HA - 6.78%), and collagen (LA - 21.16%; HA - 30.00%).

**Table 1:** Estimates of the population averages in the original sample $\left({\hat{m}}_{LA\;}e\;{\hat{m}}_{HA}\right)$ and in the 1000 bootstrap samples $\left({\hat{m}}_{LA}^{*}\;e\;{\hat{m}}_{HA}^{*}\right)$, estimates for the bias of the average ($B_{LA}^{\hat{m}}\;e\;B_{HA}^{\hat{m}})\;$and standard deviation ($B_{LA}^{s}\;e\;B_{HA}^{s}$) for chemical composition and nutritional quality variables in low ash (LA) and high ash (HA) poultry by-product meal.

Variables (Y)^1^	$\hat{m}$_LA_^2^	${\hat{m}}_{\text{LA}}^{*}$	$B_{LA}^{\hat{m}}$	$\hat{m}$_HA_^3^	${\hat{m}}_{\text{HA}}^{*}$	$B_{HA}^{\hat{m}}$	s_LA_	$s_{\text{LA}}^{*}$	$B_{LA}^{s}$^6^	s_HA_	$s_{\text{HA}}^{*}$	$B_{HA}^{s}$^7^
DM (g kg^−1^)^4^	950.91	950.88	0.035	956.88	956.81	0.064	16.63	16.49	0.135^ns^	27.73	27.57	0.153^ns^
MM (g kg^−1^)^4^	87.72	87.68	0.035	211.92	211.87	0.046	17.28	17.06	0.221^ns^	41.51	41.16	0.354^ns^
CP (g kg^−1^)^4^	735.44	735.37	0.066	634.03	633.74	0.292	37.31	37.06	0.249^ns^	46.88	46.27	0.613^ns^
EE (g kg^−1^)^4^	117.36	117.30	0.057	119.62	119.55	0.064	24.32	24.14	0.175^ns^	17.77	17.64	0.132^ns^
WA^4^	0.351	0.351	−0.000	0.3225	0.3227	−0.000	0.086	0.085	0.000^ns^	0.154	0.153	0.000^ns^
IVDOM^4^	80.27	80.27	0.002	83.16	83.19	−0.038	3.86	3.84	0.024^ns^	5.64	5.60	0.035^ns^
COL (g kg^−1^)^5^	186.04	186.04	0.003	321.18	321.81	−0.634	39.36	38.91	0.451^ns^	96.35	94.72	1.626^ns^

1 Abbreviations: COL, collagen; CP, crude protein; DM, dry matter; EE, ethereal extract; IVDOM, in vitro digestibility coefficients of OM; MM, mineral matter; WA, water activity.

2,3 Averages of the poultry by-product meal mother-samples.

4,5 Sampling size: ^4^n_LA_ = 104 and n_HA_ = 96, ^5^n_LA_ = 66 and n_HA_ = 34.

6,7 ns: nonsignificant (*P >* 0.05) by the right-hand tail chi-square test for one-sample variance $\left(H_{0}{:\;\sigma }_{\text{LA}}^{2}={\left(s_{\text{LA}}^{*}\right)}^{2}\text{or}\;H_{0}{:\;\sigma }_{\text{HA}}^{2}={\left(s_{\text{HA}}^{*}\right)}^{2}\right)$.

**Figure 2:**
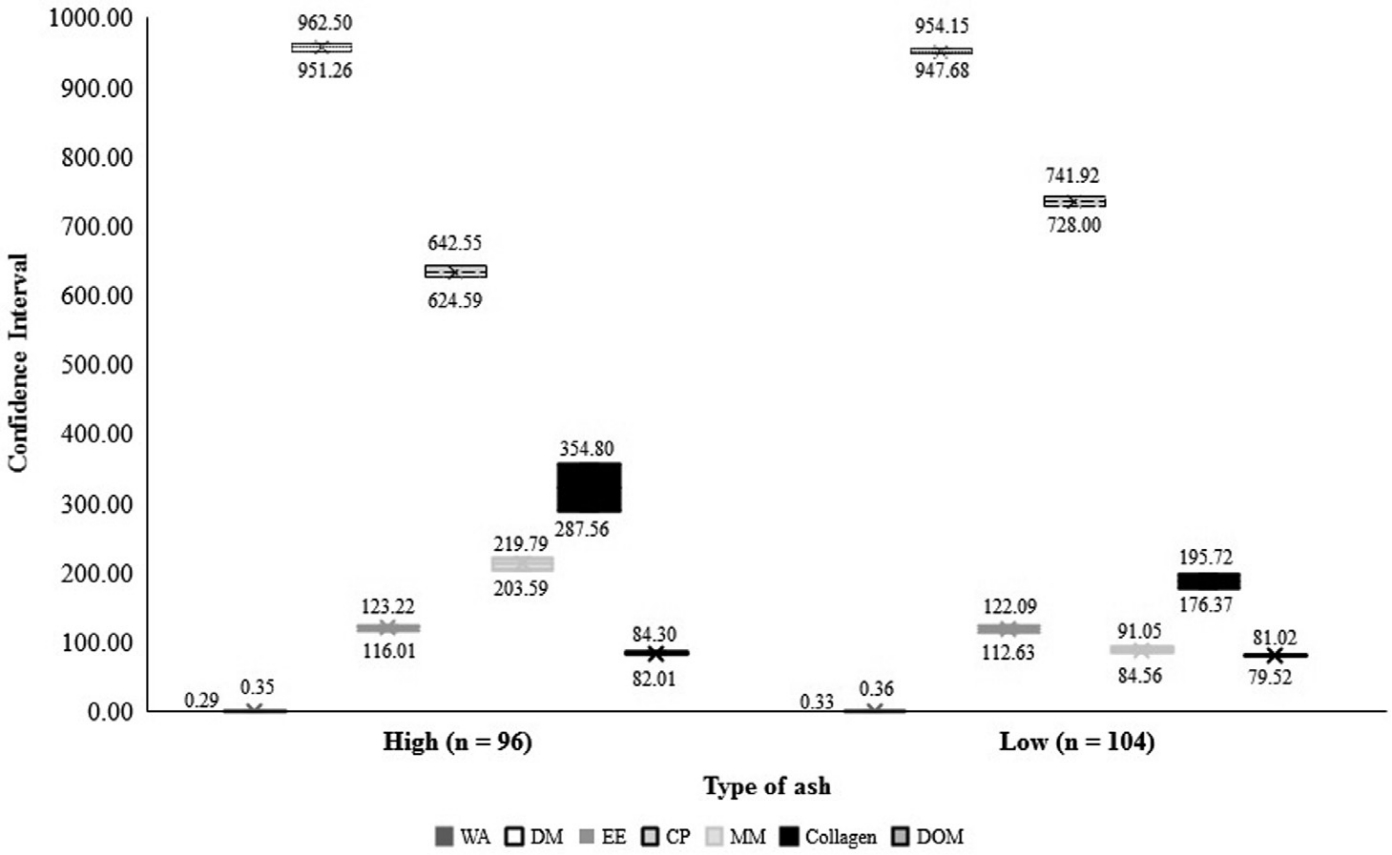
Confidence intervals (CI) of the population average estimated by different methods and significance probability of the CI of difference between averages for variables of chemical composition and nutritional quality in low ash and high ash poultry by-product meal. ^1^WA: water activity, DM: dry matter (g kg^−1^), EE: ether extract (g kg^−1^), CP: crude protein (g kg^−1^), MM: mineral matter (g kg^−1^), collagen (g kg^−1^), DOM: in vitro digestibility coefficients of organic matter (%).

In comparisons between the CI of the LA and HA meals, there was similarity (*P =* 0.451) between the PBM for the variable EE. The high ash PBM showed (*P <* 0.0001) higher DM, MM, IVDOM and collagen; however, the CP content and WA were higher (*P <* 0.0001) in the low ash PBM (Figure 2).

**Figure 3:**
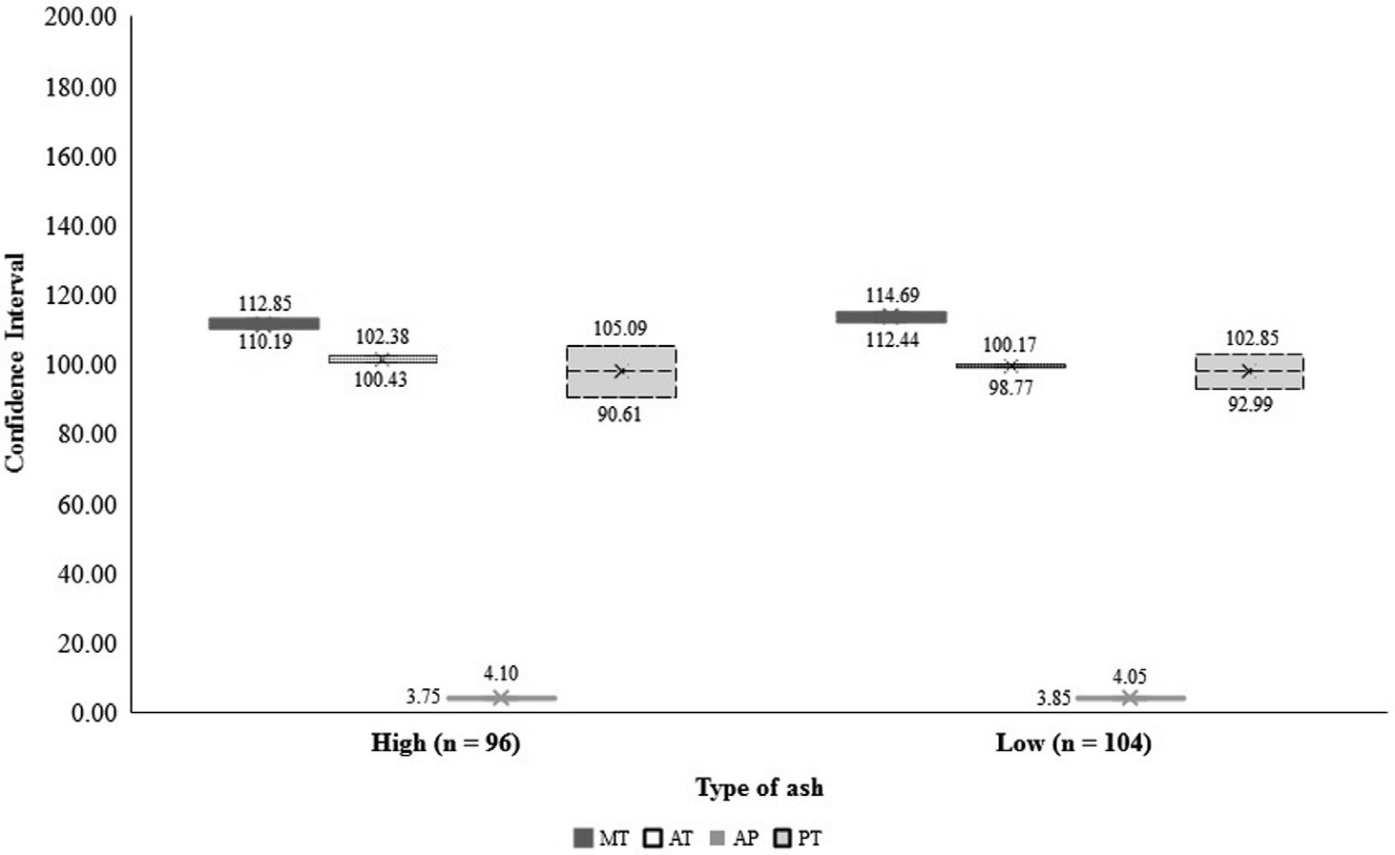
Confidence intervals (CI) of the population average estimated by different methods and significance probability of the CI of difference between averages of measured variables in low ash and high ash poultry by-product meal processing plants. ^1^MT: maximum temperature (°C), AT: average temperature (°C), AP: average pressure (kgf), PT: processing time (min).

For the process variables, the following coefficients of variation were obtained for maximum temperature (LA - 3.91%; HA - 3.56%), average temperature (LA - 3.73%; HA - 4.71%), processing time (LA - 27.37%; HA - 37.59%) and pressure (LA - 13.41%; HA - 24.23%). It can be verified by these results that the processing time showed the greatest variation in the PBM production in the establishments, reflecting directly on the CI for this variable, which also showed the greatest amplitude among all process variables involved (Table 2 and Figure 3).

**Table 2:** Estimates of the population averages in the original sample$\left({\hat{m}}_{LA\;}e\;{\hat{m}}_{HA}\right)$ and in the 1,000 bootstrap samples $\left({\hat{m}}_{LA}^{*}\;e\;{\hat{m}}_{HA}^{*}\right)$, estimates of the bias between averages ($B_{LA}^{\hat{m}}\;e\;B_{HA}^{\hat{m}}),$ estimates of the standard deviation in the original sample (S_LA_ e S_HA_) and the average standard deviation in the 1000 bootstrap samples ($s_{LA}^{*}\;e\;s_{HA}^{*})$ and estimates of the bias between standard deviation and average standard deviation $(B_{LA}^{s}\;e\;B_{HA}^{s}$) for variables measured at low ash (LA) and high ash (HA) poultry by-product meal processing plants.

Variables 1	$\hat{m}$_LA_^2^	${\hat{m}}_{\text{LA}}^{*}$	$B_{LA}^{\hat{m}}$	$\hat{m}$_HA_^3^	${\hat{m}}_{\text{HA}}^{*}$	$B_{HA}^{\hat{m}}$	s_LA_	$s_{\text{LA}}^{*}$	$B_{LA}^{s}$^6^	s_HA_	$s_{\text{HA}}^{*}$	$B_{HA}^{s}$^7^
MPT^5^ (°C)	113.36	113.35	0.001	111.64	111.65	−0.010	4.43	4.33	0.099^ns^	3.97	3.82	0.155^ns^
APT^4^ (°C)	99.48	99.46	0.016	101.44	101.42	0.028	3.71	3.67	0.042^ns^	4.78	4.76	0.027^ns^
APP (kgf) 4	3.95	3.95	0.000	3.92	3.92	0.000	0.53	0.52	0.006^ns^	0.95	0.94	0.010^ns^
PT (min) 4	97.74	97.76	−0.025	98.05	98.05	0.002	26.95	26.80	0.150^ns^	36.76	36.54	0.224^ns^

1 Abbreviations: APT, average process temperature; APP, average process pressure; MPT, maximum process temperature; PT, processing time.

2,3 Averages of the poultry by-product meal mother-samples.

4,5 Sampling size: ^4^n_LA_ = 104 and n_HA_ = 96, ^5^n_LA_ = 66 and n_HA_ = 34.

6,7 ns: non-significant (*P >* 0.05) by the right-hand tail chi-square test for one-sample variance $\left(H_{0}{:\;\sigma }_{\text{LA}}^{2}={\left(s_{\text{LA}}^{*}\right)}^{2}\text{ou}\;H_{0}{:\;\sigma }_{\text{HA}}^{2}={\left(s_{\text{HA}}^{*}\right)}^{2}\right)$.

In comparisons between the CI of LA and HA meals, the 2 types of PBM were different (*P <* 0.05) for the variables measured in the processing plants. Higher CI were observed in the low ash PBM for maximum temperature (*P <* 0.0001) and average pressure (*P <* 0.0001); however, values were greater for average temperature (*P <* 0.0001) and processing time (*P =* 0.024) in high ash PBM (Figure 3).

**Figure 4:**
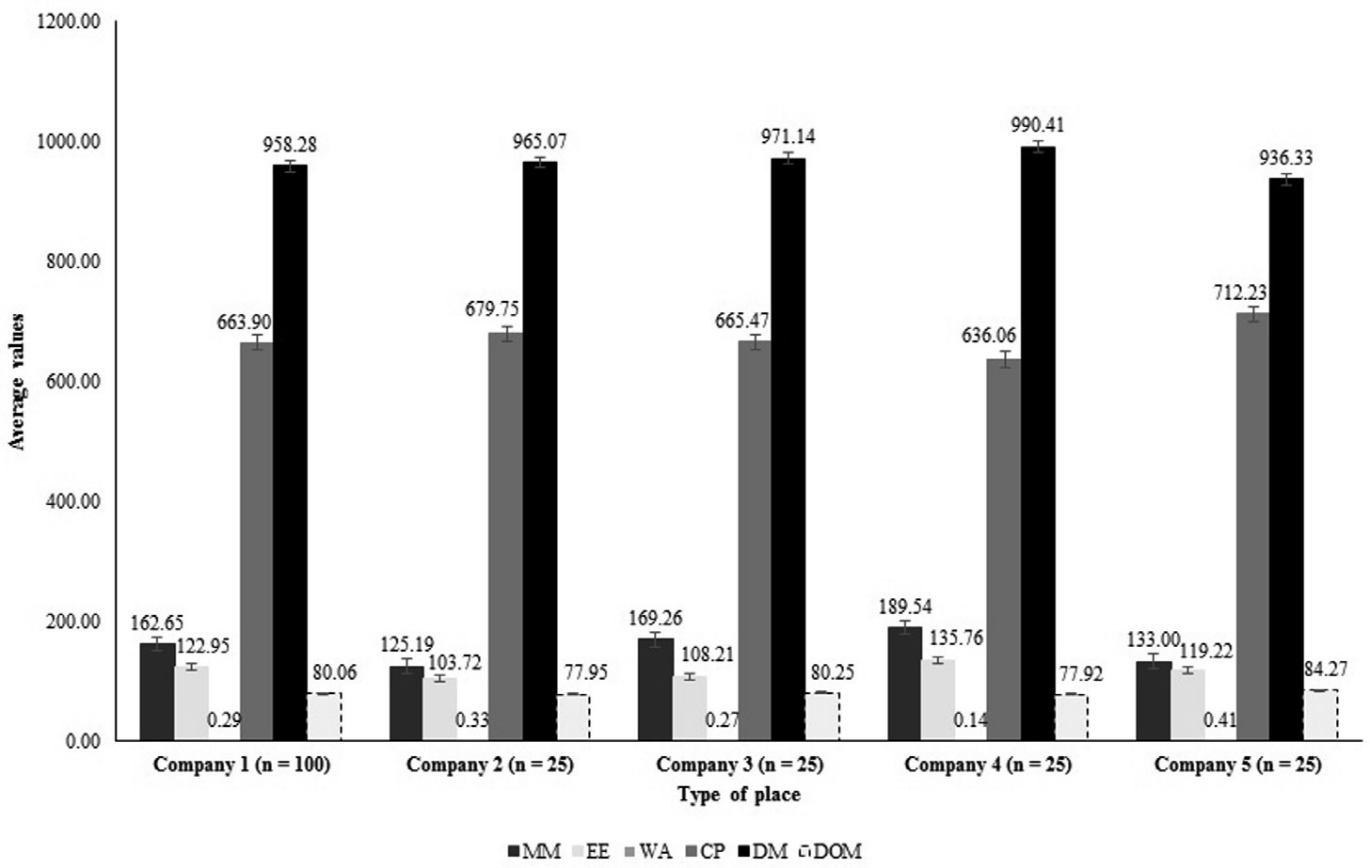
Confidence intervals of the population average for chemical composition and nutritional quality variables in poultry by-product meal within each sampled company. ^1^MM: mineral matter (g kg^−1^), EE: ether extract (g kg^−1^), WA: water activity, CP: crude protein (g kg^−1^), DM: dry matter (g kg^−1^), DOM: in vitro digestibility coefficients of organic matter (%).

The results of the present study fully demonstrated a wide variation in the chemical composition and nutritional value of PBM, as well as in the processing steps within each company (Figures 3 and 4).

In the characterization of the establishments there was an increase of 51.4% and 30.9% in MM and EE, respectively (company 4 vs. company 2). For WA, CP, DM and DOM, the results indicated an increase of 184, 11.9, 5.7, and 8.1%, respectively, between company 5 vs. company 4 (Figure 4).

**Figure 5:**
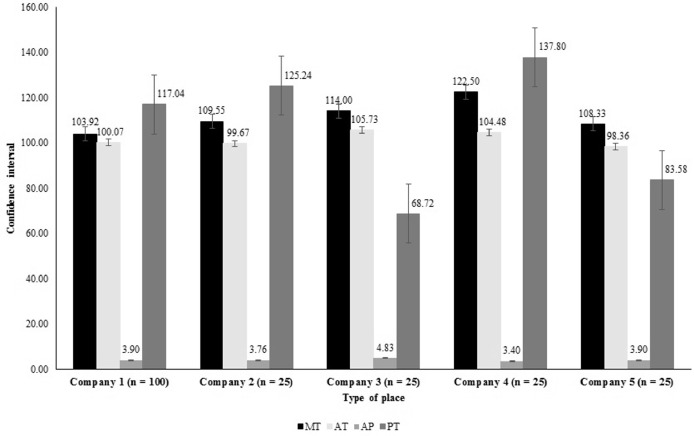
Confidence intervals of the population average for characteristics measured in the poultry by-product meal processing steps within each sampled company. ^1^MT: maximum temperature (°C), AT: average temperature (°C), AP: average pressure (kgf), PT: processing time (minutes).

These results were also verified in the processing step, where there was an increase of 17.8% for maximum temperature (company 4 vs company 1), 7.5% for average temperature (company 3 vs company 5), 42.1% for average pressure (company 3 vs company 4), and 100.5% for processing time (company 4 vs company 3) (Figure 5).

## DISCUSSION

The data from this study demonstrated variation in the chemical composition and nutritional value of commercialized PBM, as well as highlighted the importance in several steps of processing animal products. This variation in composition is a result of differences in the raw materials used and the processing system (Mcnaughton et al., 1977; Johnson and Parsons, 1997; Hassanabadi et al., 2008; Jafari et al., 2011). The DM values of all PBM indicated conformity with the results reported by Bureau et al. (1999), Dozier et al. (2003), Najafabadi et al. (2007), and Cramer et al. (2007).

These results for DM promote lower moisture and water activity (Leiva et al., 2018) because the heat treatment and pressure above 40 min used during processing of PBM eliminates microorganisms from the raw material. Although these maximum values are considered within a safe standard in meals of animal origin, according to the data obtained in the present study, while the processing produces ingredient with low moisture content observed in the average DM of 95% and relatively stable and with low variation, the coefficient of variation of water activity was one of the most varied in the current study, being important its monitoring frequently aiming at the production of safer ingredients.

For the ash content, the data showed a greater value in the PBM_HA_ samples. When comparing the maximum limit found for PBM_LA_ (91.05 g kg^−1^) with the minimum of PBM_HA_ (203.59 g kg^−1^), there was a difference of 55.27%. However, when comparing the minimum ash value of PBM_LA_ (84.56 g kg^−1^) with the maximum of PBM_HA_ (219.79 g kg^−1^) this difference increased to 61.52%.

In a study conducted by Dozier et al. (2003), who observed similar difference (48.05%) between the minimum (107 g kg^−1^) and maximum (206 g kg^−1^) ash content in PBM. Wang and Parsons (1998) reported an ash content of meat and bone meal (n = 32) ranging from 198 g to 473 g kg^−1^ in DM. Analogous results were also verified by Hendriks et al. (2002), who analyzed batches of meat and bone meal (n = 94) produced in New Zealand and found ash content ranging from 130 g to 565 g kg^−1^ in DM (µ = 283 g). These authors pointed out that the high variation in ash content observed between sources of PBM and within production plants is related to the difference in bone content of the raw material.

These ash concentrations found in this study relate directly to the collagen contents of the ingredient. The PBM_HA_ is mainly composed by residue from mechanically separated meat for the human consumption. Thus, this material presents high amount of calcium and phosphorus. Although the bone is formed by 50 to 70% of minerals, on dry matter basis, there is approximately 20 to 40% of organic matrix that supports the mineral deposition, majority formed by collagen. Due to this, minerals and collagen content are correlated in these poultry meals.

High concentrations of bone and/or connective tissue can negatively affect amino acid profile and digestibility (Oba et al., 2020). Therefore, PBM_HA_ derived from raw material rich in collagen (Pérez-Calvo et al., 2010) such as high amounts of muscle tissue (mechanically separated meat) and bone waste had greater digestibility (CI 82.01–84.30%) than PBM_LA_ (CI 79.52–81.02%). Although collagen has a low biological value due to its lower content of essential amino acids, its digestibility is high (Bindari et al., 2018), unlike in PBM_LA_, in which contains by-products derived from the organs and intestines with lower digestibility, but with a better amino acid profile.

The average estimated value for EE (119 g kg^−1^) of PBM was close to the values found by Senkoylu et al. (2005) (116 g kg^−1^), Samli et al. (2006) (118 g kg^−1^), Dozier et al. (2003) (144 g kg^−1^) and Cramer et al. (2007) (129 and 149 g kg^−1^), and below the average values observed by Najafabadi et al. (2007), Geshlog-Olyayee et al. (2011), Jafari et al. (2011), and Zarei et al. (2014), who reported results above 200 g kg^−1^. The aforementioned authors explained that these differences may be related to the starting material, added fat, or the efficiency of the equipment that extracts the fat from the cooked material. The wide variation in EE content in this study may be related to the pressing cooked material, which can be better standardized with investment in equipment and standardization in the temperature of the material entering the press.

The CP limits of 728.00 g (minimum) and 741.92 g (maximum) for the PBM_LA_ were close to those of Cramer et al. (2007), who obtained (719 g and 726 g) by evaluating the protein quality of several animal meals. On the other hand, the values of the present study were above the CP levels verified by Najafabadi et al. (2007), who assessed three PBM processing plants (565.00–634.00 g kg^−1^); however, the aforementioned authors obtained values close to the present study for PBM_HA_, in which the minimum limit was 624.59 g and the maximum was 642.55 g.

Although the process allows recovery of CP and fat from the raw material, it is also possible that the quality of the CP is negatively affected (Kawauchi et al., 2014; Zarei et al., 2014; Gooding and Meeker, 2016; Eagleson et al., 2018) because proteins tend to form insoluble portions with increasing temperature (Hicks and Verbeek, 2016) and the duration of the process can be directly influenced by the types of systems and technologies that vary between processing plants and can contribute to variation in protein quality (Meeker and Hamiltom, 2006; Jafari et al., 2011).

The estimated average for the processing time and temperature of PBM (98 min and 113°C) was lower than the time intervals (200–220 min; 170–190 min; 180–200 min) and temperatures (115°C, 135°C, and 165°C) of three different PBM processing plants verified by Jafari et al. (2011) and also below the average process temperature estimated by Pérez-Calvo et al. (2010) (141.8°C and 150°C) between 2 process plants for meat meal.

These results suggest that variation in the processing of animal meal between and within plants or production sites is attributed by the type and composition of raw material and operations control (Johnson and Parsons, 1997; Zarei et al., 2014). This variability can affect digestibility and nutrient profiles such as amino acids, making it difficult to determine protein quality (Yamka et al., 2003). The different proportions of animal tissues and parts, as well as batch uneven and individual variations between process operators and production companies affect the quality and digestibility of PBM (Laflamme et al., 2014), an observation we were able to detect in the current study.

Processing temperature and ash content are 2 indicative factors that can affect the digestibility of PBM (Hendriks et al., 2002). In a more intense processing with high temperature and pressure (135°C and 3 bar) applied to meat and bone meal with 35% CP and 50% MM, there was an increase in the digestibility of CP for cats (De Oliveira et al., 2011). Kondos and McClymont (1972) observed no reduction in amino acid availability when meat and bone meal was processed at temperatures of 121°C and 138°C.

In other research, Shirley and Parsons (2000) and Pérez-Calvo et al. (2010) also found that at temperatures at 133°C, there were no differences between PBM processing plants in terms of contribution of essential and non-essential amino acids to the CP content. However, increased processing time has been shown to decrease the digestibility and availability of amino acids (Johnson et al., 1998) and temperatures from 121°C to 126°C are sufficient to overheat proteins and damage the amino acid profile (Pesti et al., 1986), impairing protein metabolism in animals (Wang and Parsons, 1998).

The negative effect of the process on the digestibility of CP can be explained by the denaturation of the protein chain structure due to burning in prolonged processing of 180 min (Piva et al., 2001), favoring racemization or cross-linked of amino acid residues (Shirley and Parsons, 2000). However, although IVDOM showed an effect on the confidence interval between PBM, the averages of the characteristics measured in the PBM processing plants were not explained by the variations in digestibility.

The raw materials vary in visceral amount, bone material, nutritional content, making it difficult to measure and identify the degree of interference of these variations in the process in each batch of raw material released in the cooker. Thus, it is important to verify the ideal process time, temperature and pressure for these batches of raw material. The segregation of these materials would be a great limitation for industries that work with poultry by-products and makes it difficult to verify the limits that these factors begin to promote harmful effects on the quality of PBM.

Based on the criteria assessed in this study, this study evidenced that the corrective measures by limiting the amount of bones in the raw material, optimizing the pressing step for the poultry fat extraction, and also controlling the processing time of PBM may favor the production of more standardized PBM in terms of chemical composition and quality. Studies with the segregation of the material that enters the production of PBM, automation of cookers, adjustments in the pressing process and formulation of meals by mixing batches of finished product can be alternatives in search of obtaining better standardization of this ingredient.

## DISCLOSURES

None of the authors of this paper have a financial or personal relationship with other people or organizations that could inappropriately influence or bias the content of the paper. All authors declare that they have no competing interests. All data generated or analyzed during this study are available from the corresponding author upon reasonable request.

## Appendix Supplementary materials

Image, application 1
